# Echocardiographic parameters in COVID-19 patients and their association with ICU mortality: a prospective multicenter observational study

**DOI:** 10.1186/s13089-023-00336-3

**Published:** 2023-09-13

**Authors:** Amarja Ashok Havaldar, Merugu Vinay Kumar, Raman Kumar, Surya Prakash Yarramalle, Mohammad Saif Khan, Krushna Chandra Misra, Shubhangi Kamble, Atul Sangale, Jay Prakash, Munta Kartik, Sumithra Selvam

**Affiliations:** 1 Department of Critical Care Medicine, St John’s Medical College Hospital, 1st floor, MICU, Bangalore, 560034 India; 2 Department of Critical Care Medicine, Rajendra Institute of Medical Sciences, Ranchi, 834009 India; 3 Department of Critical Care Medicine, Yashoda Hospital, Somajiguda, 500082 Hyderabad India; 4 Department of Critical Care, Apollo Hospital, Nashik, 422003 India; 5 Department of Epidemiology and Biostatistics, St Johns Research Institute, Bangalore, India 560034

**Keywords:** COVID-19, Diastolic dysfunction, Echocardiography, Intensive care unit (ICU), Systolic dysfunction, Troponin I, Ventricular dysfunction

## Abstract

**Background:**

Echocardiography has become an integral part of the management of critically ill patients. It helps to diagnose and treat various conditions. COVID-19 patients can develop cardiac dysfunction. We planned to study the echocardiographic parameters in COVID-19 patients.

**Methods:**

We conducted a prospective observational multicenter study after institutional ethical committee approval. COVID-19 pneumonia patients admitted to the intensive care unit (ICU) were enrolled. The echocardiographic evaluation was done within 24–48 hours of admission. Assessment of the left and right heart with systolic and left ventricular diastolic function evaluation was done. The primary outcome was ICU mortality. The secondary outcomes were the length of ICU stay and duration of mechanical ventilation.

**Results:**

Among 573 patients mean age was 57.17 (14.67) with 68.60% being males. On day 1 of ICU, invasive mechanical ventilation was used in 257 (45%) patients. One hundred and forty-eight (25.83%) patients were on vasopressors when echocardiography was performed. Severe left ventricle (LV) systolic dysfunction was seen in 8.7% of patients and had higher odds of mortality [2.48(1.058–5.807), *p = *0.037] followed by E and e′ with odds ratio of [0.984(0.971–0.998), *p = *0.021] and 0.897 (0.805–0.998), *p = *0.046], respectively. E/e′ indicative of filling pressure of the LV was not found to be significant. Troponin I, E/A, and RV dilatation were similar among survivors and non-survivors.

**Conclusion:**

Echocardiographic evaluation in COVID-19 patients showed severe LV systolic dysfunction was associated with ICU mortality. E/e′ was not found to be significant but lower e′ was associated with higher mortality.

*Trial registration* IEC 131/2020, CTRI/2020/06/025858 date 13th June 2020

## Background

Echocardiography plays a vital role in diagnosing and treating critically ill patients. Majority of the hospitals use echocardiographic evaluation as an initial screening tool. Echocardiography has various advantages such as availability at the bedside, real-time evaluation, and repeatability as and when necessary [1, 2]. Assessment of COVID-19 patients is not an exception to this.

During the pandemic, it was difficult to perform detailed echocardiography and the focus was shifted to point-of-care echocardiography [3]. Earlier studies have reported difficulties in echocardiographic assessment in acute respiratory distress syndrome (ARDS) patients [4–6]. Technical challenges in image acquisition for mechanically ventilated patients, the effect of positive end-expiratory pressure (PEEP) on echo parameters, and apprehension among the healthcare workers in using ultrasound during COVID-19 were some of the difficulties [4–6].

The global survey from 63 countries studied confirmed or suspected COVID-19 patients and showed cardiac abnormalities in 50% of patients [7]. There are various studies describing systolic dysfunction of the right and left ventricles and biomarkers [8, 9]. Huang et al. observed LV and RV (right ventricle) systolic dysfunction in one-third of COVID-19 patients. Acute cor pulmonale and age were the predictors of ICU and hospital mortality [8]. Jansson et al. showed COVID-19 patients who developed acute myocardial injury diagnosed with elevated high sensitivity troponin I (hsTnT), a small subset of these patients had LV and RV dysfunction [9]. There is limited data on the assessment of diastolic dysfunction in COVID-19 patients [10, 11]. We aimed to evaluate the association between the echo parameters (right and left ventricular systolic function, LV diastolic function including Troponin I), and ICU mortality.

## Methods

A prospective observational study across the 4 centers was conducted. Institutional ethical committee approval was obtained (IEC 131/2020). The echocardiographic evaluation was a routine practice in the majority of ICUs. The waiver of consent was given for all the centers except one. This is a detailed echocardiographic assessment of EPIC19 study patients [12]. Patients were admitted to the ICU for the need for organ support like ventilation, vasopressor, renal replacement therapy, neuromonitoring, or anticipated worsening of the clinical condition. The admission criteria were as per the treating physician. Patients in whom echocardiographic evaluation was done were included in the study. Patients with poor echo window were excluded. Strengthening the Reporting of Observational Studies in Epidemiology (STROBE) guidelines were followed. The inclusion criteria were laboratory-confirmed cases of COVID-19 ICU patients. The primary outcome was ICU mortality. The secondary outcomes were the length of ICU stay and duration of mechanical ventilation.

The transthoracic echocardiography (TTE) was performed with Sonosite Edge II or Philips HD11XE machine within 24–48 hours of ICU admission. The cardiac or phased array probe of 2.5 MHz was used. 2D (2 dimensional) echocardiography was done by the intensivists who have received training in focused echocardiography and ultrasound and who are routinely performing screening echocardiography. The majority of ICUs are teaching hospitals hence the echocardiographic evaluation was done by the trainees and supervised by the senior faculty. The echocardiographic evaluation included looking for the heart chamber enlargement and assessment of systolic and diastolic function. The systolic function was assessed by the visual gestalt method. This method evaluates systolic function qualitatively based on the endocardial thickening and also looks into regional wall motion abnormalities. The heart function was assessed by eyeballing using an apical four-chamber view (A4C), parasternal short and long axis, or subcostal view. Left ventricular systolic function was classified as hyperdynamic, good, mildly reduced, moderately reduced, and severely reduced. The presence of any regional wall motion abnormalities or dilatation of the left or right ventricle was captured based on the qualitative evaluation. Right ventricular systolic function was assessed by tricuspid annular pulmonary systolic excursion (TAPSE). RV dysfunction was defined as TAPSE < 1.7 cm [13].

The diastolic function was evaluated by transmitral flow velocity. Early diastolic wave (E) due to rapid filling of the left ventricle (LV), and late systolic wave (A) due to late filling of LV were measured. E/A was calculated. The mitral annular flow velocities were measured. The diastolic waves e′ and a′ were measured at the lateral mitral annulus. E/e′ indicative of the filling pressure of the LV was calculated [8, 9]. We used lateral e′ for calculation for the uniform data capture. For patients with mitral valve pathology or having atrial fibrillation, a doppler assessment was not performed. The classification of diastolic dysfunction was done based on the lateral e′ velocity of < 10 cm/s and E/e` values further categorized based on the E/e′ [14, 15].

The E/e′ of < 8, E/e′ 8–12, and E/e′ ratio > 12 are graded as grade I, II, and III. It was based on the study by Lanspa, et al [16]. As we measured lateral e′ velocity, we used standard cut-offs applicable to lateral e′ [15, 16]. We reported data according to the PRICES statement mentioning (1) baseline characteristics and comorbidities; (2) vasopressors requirement and need for invasive ventilator support, plateau pressure, and positive end-expiratory pressure (PEEP); (3) information on LV systolic, RV systolic, and LV diastolic function including biomarker Troponin I [17].

## Statistics

The mean (standard deviation SD), or median (interquartile range IQR) were used as indicated. The categorical variables were presented as (%) percentages. The quantitative data with parametric and nonparametric distribution were analyzed with the ‘Independent *t* test’ and ‘Mann–Whitney *U*’ test, respectively. The Chi-square test was used for qualitative data analysis. To assess the association between various echocardiographic parameters with ICU mortality, considering the collinearity, each echocardiographic parameter was analyzed separately. Univariate and multivariable logistic regression analysis was performed. Clinical relevance and variables that had a *p*-value less than 0.10 in the univariate analysis were considered for multivariable logistic regression and an adjusted *p-*value less than 5% was considered statistically significant. We used STATA 15, College Station, TX software.

## Results

Among 667 patients, 30 patients with poor echo window were excluded as per the exclusion criteria, and for 64 (10.04%) patients data were unavailable. Total of 573 patients were included with 459, 48, 35, and 31 patients from each center, and 95% of echo evaluation was performed within 24 hours of ICU admission. The mean age was 57.17 (14.67) and 68.60% were males. The Acute Physiology, Age, and Chronic Health Evaluation (APACHE II) score was 30.10 (5.96) and the Sequential Organ Failure Assessment (SOFA) score was 7 (4–11). Two seventy-six patients (48.16%) were transferred from the ward to ICU. Among these noninvasive ventilation (NIV) was used in 82 (29.71%) patients before ICU admission. On day1 of ICU, invasive mechanical ventilation was used in 257 (45%), NIV in 148 (25.83%), oxygen therapy in 141 (24.60%), HFNC in 15 (2.62%), and 12 (2.09%) patients were on room air. One hundred and forty-eight (25.83%) patients were on vasopressors when echocardiography was performed. Electrocardiogram (ECG) on day 1 was recorded. The predominant ECG rhythm was sinus. The mean heart rate was 97 (20) beats per minute on day 1 of ICU. The maximum PEEP (PEEPmax) was 10.52 (3.43) cm of H_2_O. Maximum plateau pressure was 30.46 (6.53) cm of H_2_O. Among the comorbidities, diabetes mellitus (DM) and ischemic heart disease (IHD) were significantly associated with mortality (Table 1). The elderly population and patients with higher APACHE II and SOFA scores were associated with mortality (*p < *0.001). Requirement of invasive ventilation support, vasopressors, and PEEPmax were significantly higher in non-survivors (*p < *0.01) (Table 1).

**Table 1:** Baseline and ventilation characteristics

Parameter	N	All	Survivors (n=233)	Non-survivors (n=340)	*p* value
Age^¥^	573	57.71 (14.67)	55.13 (14.46)	59.47 (14.57)	< 0.001
Gender	573	M/F 393/180	M/F (165/68)	M/F (228/112)	0.341
		(68.59/31.41)	(70.82/29.18)	(67.06/32.94)	
History of DM	573	331 (57.77)	123 (52.79)	208 (61.18)	0.046
History of hypertension	573	338 (58.99)	127 (54.51)	211 (62.06)	0.071
History of IHD	573	85 (14.83)	22 (9.44)	63 (18.53)	0.003
History of CVA	573	40 (6.98)	9 (3.86)	31 (9.12)	0.015
History of CKD	573	80 (13.96)	27 (11.59)	53 (15.59)	0.175
History of COPD	573	24 (4.19)	8 (3.43)	16 (4.71)	0.455
APACHE II	525	30.10 (5.96)	28.39 (5.85)	31.23 (5.78)	< 0.001
SOFA	461	7 (4–11)	5 (3‑7)	8 (5–12)	< 0.001
Invasive ventilation	257	257 (44.85)	63 (27.04)	194 (57.06)	< 0.001
Vasopressors	148	148 (25.80)	47 (20.2)	101 (29.70)	0.010
Heart rate	573	97 (21)	93 (20)	99 (22)	< 0.001
PEEP max (cm of H_2_O)	397	10.52 (3.43)	9.70 (3.21)	10.87 (3.47)	< 0.001

Values are n (%), p value from Chi-square test of association

^**¥**^Mean (SD), independent sample *t*-test was used for comparison. ^**§**^ Median (IQR), Mann–Whitney *U* test.* DM* Diabetes Mellitus,* IHD* Ischemic heart disease,* CVA* Cerebrovascular accident,* CKD* Chronic kidney disease,* COPD* Chronic obstructive pulmonary disease.

### Outcomes

The ICU mortality was 60% (95% CI 55–63%). The secondary outcomes were similar between survivors and non-survivors (Table 2).

**Table 2:** Primary and secondary outcomes

	*N*	All	Survivors	Non-survivors	*p* value
**Primary outcome**					
ICU mortality	573	573	233 (40%)	340 (60%)	
**Secondary outcomes**					
Length of ICU stay^§^	573	7 (4–13)	6 (4–11)	8 (4–14)	0.138
Duration of mechanical ventilation^§^	349	7 (3–12)	7 (5–10)	6 (3–12)	0.305

Values are *n* (%), *p* value from Mann–Whitney *U* test. ^§^ Median (interquartile range)

### Echocardiographic evaluation

The systolic dysfunction was classified into different categories (Table 3). Severe systolic dysfunction was significantly associated with mortality (*p = *0.007). The regional wall motion abnormalities (RWMA) were higher in non-survivors than survivors (*p = *0.005) (Table 3). Among patients having RWMA (90 patients), 85.55% of patients had LV systolic dysfunction.

**Table 3:** Echocardiography parameters

Echoparameters	*N*	All	Survived *n* = 233	Non-survivor *n* = 340	*P*-value
**LV systolic function (Visual gestalt method)**	540				
Hyperdynamic		12 (2.22)	2 (0.94)	10 (3.05)	0.007
Good		341 (63.15)	142 (66.98)	199 (60.67)	
Mildly reduced		47 (8.70)	21 (9.91)	26 (7.93)	
Moderately reduced		93 (17.22)	39 (18.40)	54 (16.46)	
Severely reduced		47 (8.70)	8 (3.77)	39 (11.89)	
RWMA	506	90 (17.79)	24 (12.24)	66 (21.29)	0.010
**LV diastolic function**					
E^¥^ (cm/sec)	215	79.43 (23.12)	83.51 (21.89)	76.60 (23.60)	0.030
A^¥^ (cm/sec)	202	75.83 (23.11)	71.02 (21.81)	79.18 (23.49)	0.013
E/A^§^	202	1.01 (0.78–1.30)	1.14 (0.94–1.42)	0.91 (0.70–1.14)	< 0.001
e′ (cm/Sec)	208	10.27 (2.95)	10.84 (2.75)	9.88 (3.03)	0.021
E/e′^§^	207	8.09 (2.65)	8.05 (2.59)	8.11 (2.69)	0.872
LV dilatation	573	251 (43.80)	109 (46.78)	142 (41.76)	0.235
RV dilatation	358	61 (17.04)	21 (15)	40 (18.35)	0.411
TAPSE (cm)	26	2.2 (1.8–2.4)	2.3 (1.8–2.56)	2.12 (1.7–2.3)	0.263
Troponin ^§^ (ng/ml)	281	0.06 (0.01–0.40)	0.03 (0.01–0.24)	0.07 (0.02–0.51)	0.015

Values are *n* (%), *p* value from Chi-square test of association. ^**¥**^Mean (SD), independent sample *t*-test was used for comparison. ^**§**^Median (IQR), *p* value from Mann–Whitney *U* test,* RWMA* Regional wall motion abnormality,* LV* Left ventricle ,* RV* Right ventricle,* TAPSE* Tricuspid annular plane systolic excursion

**Fig. 1.**
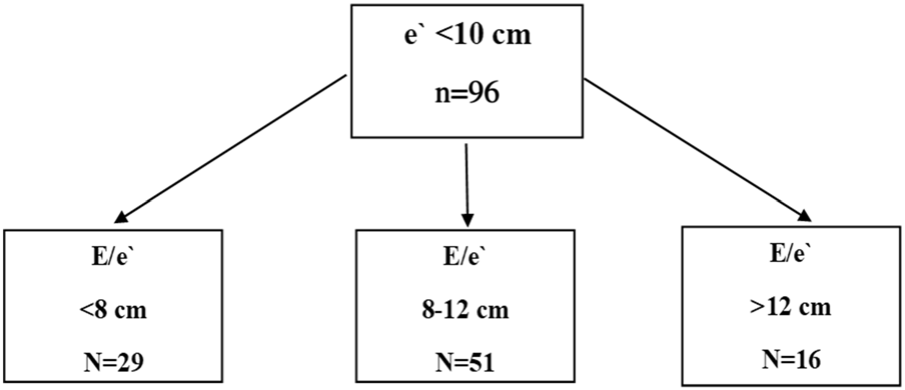
Diastolic dysfunction classification based on e` and E/e`

The diastolic function evaluation was routinely performed in one of the centers [18]. It showed significantly lower E/A values among non-survivors than in survivors [0.91 (0.70–1.14) vs 1.14 (0.94–1.42), *p < *0.01]. The e′ indicative of relaxing properties of the LV was significantly lower in non-survivors than in survivors [9.88 (3.03) vs 10.84 (2.75) vs, *p = *0.021]. The E/e′ was not a significant parameter (Table 3). Grade I, II, and III diastolic dysfunction was seen in 30%, 53%, and 17%, of patients, respectively, was not statistically significant (*p = *0.562) (Fig. 1). Troponin I was significantly higher in non-survivors than survivors. (*p = *0.015) (Table 3).

We did a logistic regression analysis of the echocardiographic parameters after adjusting for the variables like age, gender, history of DM, hypertension, IHD, need for invasive ventilator support, and requirement of vasopressors (Table 4). The adjusted odds ratio showed severe LV systolic dysfunction, E and e′ were the significant parameters predictive of mortality with the odds ratio of 2.48 (1.058–5.807), 0.984 (0.971–0.998) and 0.897 (0.805–0.998), respectively.

**Table 4:** Unadjusted and adjusted odds ratio of the echocardiographic parameters with ICU mortality

Parameters	Unadjusted odds ratio (95% CI)	*P* value	Adjusted odds ratio (95% CI)	*P* value
LV systolic dysfunction				
Hyperdynamic	3.56 (0.77–16.53)	0.104		
Mild	0.88 (0.48–1.63)	0.692		
Moderate	0.98 (0.62–1.57)	0.959		
Severe	3.48 (1.57–7.66)	0.001	2.48 (1.058–5.807)	0.037
LV dilatation	0.816 (0.58–1.141)	0.235	0.707 (0.486–1.029)	0.070
E	0.987 (0.975–0.999)	0.033	0.984 (0.971–0.998)	0.021
A	1.016 (1.003–1.029)	0.015	1.012 (0.998–1.027)	0.085
E/A	0.628 (0.387–1.020)	0.060	0.650 (0.399–1.059)	0.083
e′	0.894 (0.812–0.984)	0.023	0.897 (0.805–0.998)	0.046
E/e′	1.029 (0.948–1.118)	0.489	1.010 (0.930–1.098)	0.811
RWMA	1.939 (1.168–3.216)	0.010	1.643 (0.941–2.870)	0.081
RV dilatation	1.273 (0.71–2.26)	0.412	1.306 (0.703–2.425)	0.398

CI confidence interval. Multivariable analysis was adjusted for age, gender, comorbidities (diabetes mellitus, hypertension and ischemic heart disease), need for ventilator support and vasopressors

## Discussion

In COVID-19 patients among the various echocardiographic parameters, severe LV systolic dysfunction was associated with ICU mortality and had a higher odds ratio (Table 4). A recent study describing patients with sepsis and septic shock showed an association of LV systolic dysfunction with hospital mortality. It showed a “U” shaped association suggesting patients with severe systolic dysfunction with ejection fraction of < 25% as well as hyperdynamic LV function with EF > 70% had higher hospital mortality [19].

The echocardiographic evaluation is feasible in patients of ARDS requiring invasive ventilator support. During COVID-19 pandemic, initial ultrasound evaluation helped to understand the disease severity better [20]. However, we did not collect information on changes in the treatment plan based on the echocardiography. In our study, 45% of the patients were on invasive ventilator support. The echocardiographic assessment was done by the intensivists (94.44%, 543/573) as against cardiologists in the Szekeley et al. study [4, 5, 10].

There are various studies describing echocardiographic parameters in COVID-19 patients. The initial study by Ceriani, et al., described elevated systolic pulmonary artery pressure as one of the significant parameters in patients with severe pneumonia [30.67 (5.16) vs 26.24 (4.34), *p = *0.006] but it was not associated with the adverse outcomes. This study used mortality and or the need for invasive ventilation as the adverse outcomes [21]. Schott et al., mentions severe RV dilatation observed in non-survivors was not statistically significant [5].

A systematic review describing echocardiographic parameters observed normal echocardiographic findings in 50% of the patients with preserved LV ejection fraction [22]. The patient population described was a general population irrespective of the severity of the illness. In our study, the ICU population was included. In the ECHO-COVID study, the majority of the patients had normal LV systolic function with abnormal LV and or RV dysfunction in one-third of patients [8]. Similarly in our study, LV systolic function was normal in 63.15% of patients. The proportion of patients having severe LV dysfunction was similar in our study 8.7% vs 6.5% in the ECHO-COVID study.

We observed LV dilatation was more prevalent (17.04%) than in the ECHO-COVID study (8%) and among the patients with septic shock requiring vasopressors (148, 25.82%), LV dilatation was observed in 47.30%. LV dilatation present in shock patients was pre-existing as acute dilatation of the left ventricle in shock is rare based on the available literature [23]. However, repeat echocardiographic assessment after the resolution of shock would have helped to confirm the pre-existing vs acute reversible LV dilatation as a result of viral myocarditis [24, 25].

In a study by Szekeley et al., systolic function was preserved and 3 patients were on vasopressor support [10]. As against in our study although the majority of the patients had normal LV systolic function, the proportion of patients having systolic dysfunction were higher (36.85%, 199/540) with 25.82% being on vasopressors. In comparison with the LV diastolic function, our patients had a lower mean E/e′ ratio (8.09 vs 10.5). This could be due to the effect of PEEP on LV preload and afterload. Also among the comorbidities, we had a lesser number of patients with IHD 14.3% as against 23% (including IHD 16%, and congestive heart failure 7%) in Szekeley’s study.

The study by Luigi La via et al. describes a single-center experience on the diastolic function evaluation in 35 patients. It showed non-survivors had lower ‘s’ wave and higher E/e′ measured at the lateral mitral leaflet [11]. As against we did not find any difference in E/e′ in non-survivors and survivors [8.11 (2.69) vs 8.05 (2.59), *p = *0.872]. We found e′ was lower in non-survivors than survivors [9.88 (3.03) vs 10.84 (2.75), *p = *0.021], suggesting impaired LV relaxation was associated with the non-survivors.

There are studies describing different phenotypic patterns of RV, classified into 3 types as preserved RV function, dilatation of RV with preserved systolic function, and class 3 as RV dilatation with severely impaired systolic function. It was a single-center retrospective study [26]. In our study, evaluation of RV systolic function by TAPSE was available only in 26 patients (Table 3).

The strengths of our study include, it is one of the few prospective multicenter studies describing biventricular echocardiographic parameters. The study describes both systolic as well as diastolic function assessment of the LV and limited information on RV systolic function and Troponin I in COVID-19 patients.

The limitations of the study are only a single echocardiographic evaluation was performed at the time of admission. Hence the subsequent effect of PEEP and different therapeutic maneuvres such as recruitment or proning could not be studied. We suggest monitoring the trend of RV function will help in implementing therapeutic strategies such as proning or extracorporeal membrane oxygenation (ECMO) as the early interventions. The echocardiography was performed by different operators, hence the interobserver variability could not be ruled out. With the training experience of the operators, only screening evaluation was possible during the pandemic. We did not include the left atrial volume index and tricuspid regurgitation velocity parameters as suggested by the 2016 guidelines [27]. The left atrial volume index will have limited applicability as an acute increase in diastolic pressure may not result in dilatation of the left atrium [28]. We used Doppler-based parameters for diastolic dysfunction evaluation (E/A, e′ at the lateral mitral annulus, and E/e′) based on the study by Lanspa et al. [16]. We strongly believe that qualitative evaluation is rapid and can provide valuable information, as compared to quantitative evaluation in ICU patients [29]. Although there are limitations with the use of qualitative evaluation, it is a rapid screening tool for ICU patients when clinicians have varied skills in echocardiography such as basic to advanced training. Echocardiographic parameters need careful interpretation for each patient’s clinical characteristics such as age, gender, comorbidities, invasive ventilation, and fluid status for deciding patient management. Ultrasound has a vital role in the management of COVID-19 patients [30]. Integration of the lung ultrasound with echocardiographic parameters and diaphragm will be useful in understanding disease severity and deciding patient management as suggested by Dell’Aquila, et al [31]. In our study, lung ultrasound was performed in one of the centers and lung ultrasound score was similar in survivors and non-survivors [12].

Future studies describing serial echocardiographic assessment are required. To observe the effect of PEEP on routine echocardiographic parameters and ventricular interdependence as well as on ventilator parameters (PaO_2_/FiO_2_ ratio, PCO_2,_ and pH) will be useful. Patients with pre-existing LV diastolic dysfunction with elevated LV filling pressure can develop ARDS due to lung pathology. Diastolic function assessment is as important as systolic function. It can help in optimization of the PEEP and fluid balance in managing interstitial and hydrostatic edema for these patients and can assist in weaning [32]. We need a comprehensive approach in managing ARDS patients. Evaluation of cardiac, lung, and diaphragm ultrasound can help in managing these patients in different phases of illness and improving outcomes [33].

## Conclusion

The study describing echocardiographic parameters in COVID-19 patients showed that left ventricular systolic dysfunction assessed by the visual gestalt method is one of the parameters to predict the ICU mortality.

## Data Availability

Data will be available after the reasonable request.

## Abbreviations

APACHE II: Acute Physiology, Age, and Chronic Health Evaluation
ARDS: Acute respiratory distress syndrome
CKD: Chronic kidney disease
COPD: Chronic obstructive pulmonary disease
CVA: Cerebrovascular accident
DM: Diabetes Mellitus
ECMO: Extracorporeal membrane oxygenation
ICU: Intensive care unit
IEC: Institutional ethical committee
IHD: Ischemic heart disease
LV: Left ventricle
PEEP: Positive end-expiratory pressure
RV: Right ventricle
RWMA: Regional wall motion abnormalities
SOFA: Sequential Organ Failure Assessment
STROBE: Strengthening the Reporting of Observational Studies in Epidemiology
TAPSE: Tricuspid annular plane systolic excursion
